# Posteriorly migrated thoracic disc herniation: a case report

**DOI:** 10.1186/1752-1947-7-41

**Published:** 2013-02-12

**Authors:** Naohisa Miyakoshi, Michio Hongo, Yuji Kasukawa, Yoshinori Ishikawa, Yoichi Shimada

**Affiliations:** 1 Department of Orthopedic Surgery, Akita University Graduate School of Medicine, 1-1-1 Hondo, Akita, 010-8543, Japan

**Keywords:** Intervertebral disc herniation, Posterior migration, Thoracic spine

## Abstract

**Introduction:**

Posterior epidural migration of thoracic disc herniation is extremely rare but may occur in the same manner as in the lumbar spine.

**Case presentation:**

A 53-year-old Japanese man experienced sudden onset of incomplete paraplegia after lifting a heavy object. Magnetic resonance imaging revealed a posterior epidural mass compressing the spinal cord at the T9-T10 level. The patient underwent emergency surgery consisting of laminectomy at T9-T10 with right medial facetectomy, removal of the mass lesion, and posterior instrumented fusion. Histological examination of the mass lesion yielded findings consistent with sequestered disc material. His symptoms resolved, and he was able to resume walking without a cane 4 weeks after surgery.

**Conclusions:**

Pre-operative diagnosis of posterior epidural migration of herniated thoracic disc based on magnetic resonance imaging alone may be overlooked, given the rarity of this pathology. However, this entity should be considered among the differential diagnoses for an enhancing posterior thoracic extradural mass.

## Introduction

Posterior epidural migration of a herniated thoracic disc is extremely rare but may occur in the same manner as it does in the lumbar spine. To the best of our knowledge, only five cases of posteriorly migrated thoracic disc herniation have been reported in the English-language literature [1-5]. We present herein an additional case of posteriorly migrated thoracic disc herniation. Differential diagnoses for this rare entity are also discussed.

## Case presentation

**Figure 1:**
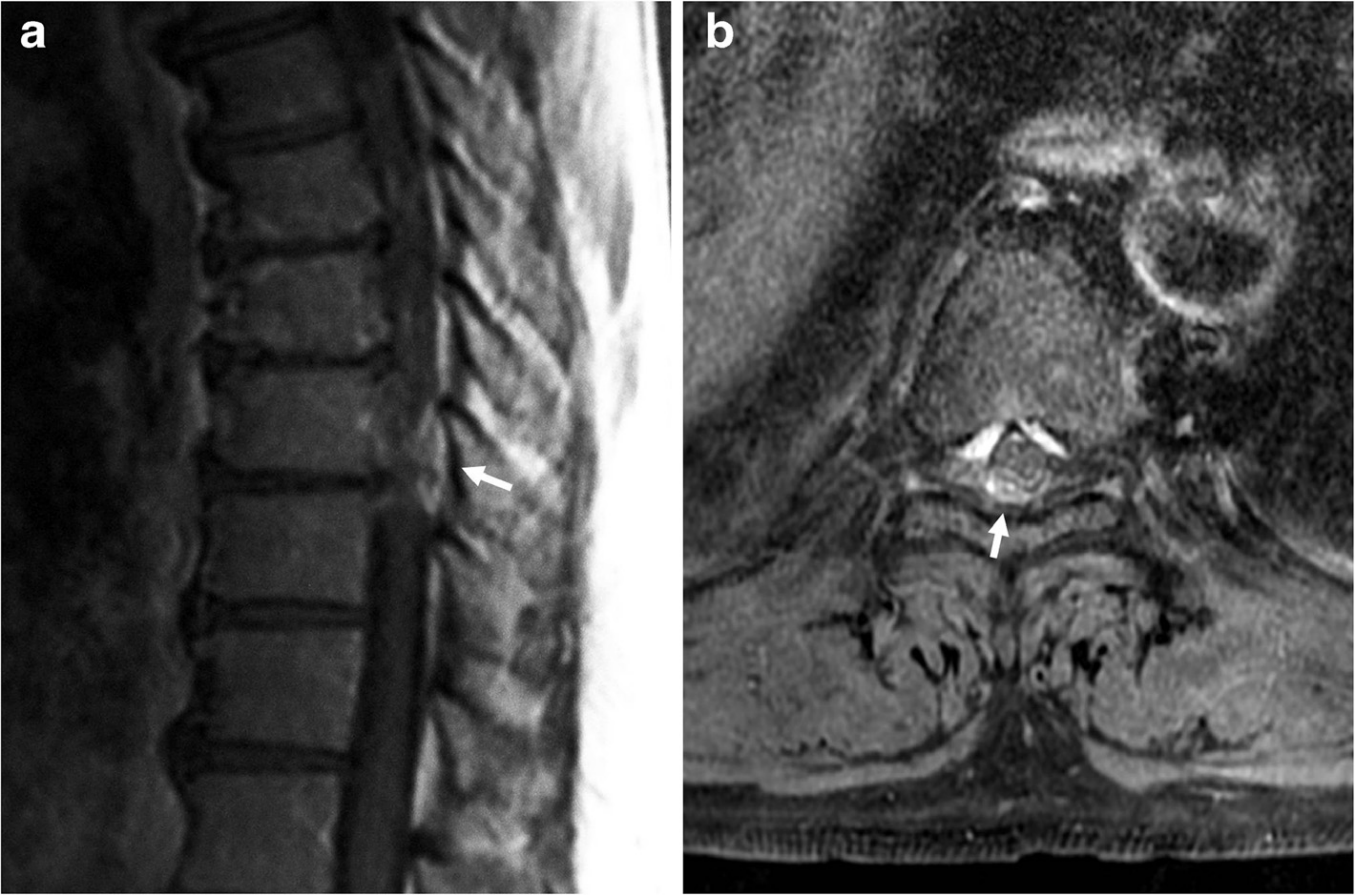
**Pre-operative magnetic resonance imaging of the thoracic spine. **Sagittal gadolinium-enhanced T1-weighted imaging **(a)** and axial gadolinium-enhanced T1-weighted imaging with fat suppression **(b)** show a peripherally and heterogeneously enhancing posterior epidural mass at the T9-T10 level, compressing the spinal cord (arrows).

A healthy 53-year-old Japanese man experienced sudden onset of incomplete paraplegia after lifting a heavy object. The physical examination revealed that his motor strength was grade 0/5 in the right lower extremity and 0–2/5 in the left lower extremity. Sensory loss was observed below the level of the umbilicus. Urinary incontinence was also present. Magnetic resonance imaging (MRI) revealed a posterior epidural mass compressing the spinal cord at the T9-T10 level (Figure 1). Compared to the spinal cord, the mass lesion appeared isointense on T1-weighted imaging, so detecting the mass with T1-weighted imaging alone would have been difficult. The mass showed slight signal hyperintensity on T2-weighted imaging but was clearly detected after gadolinium administration, which showed peripheral contrast enhancement. Blood tests showed no abnormalities. 

**Figure 2:**
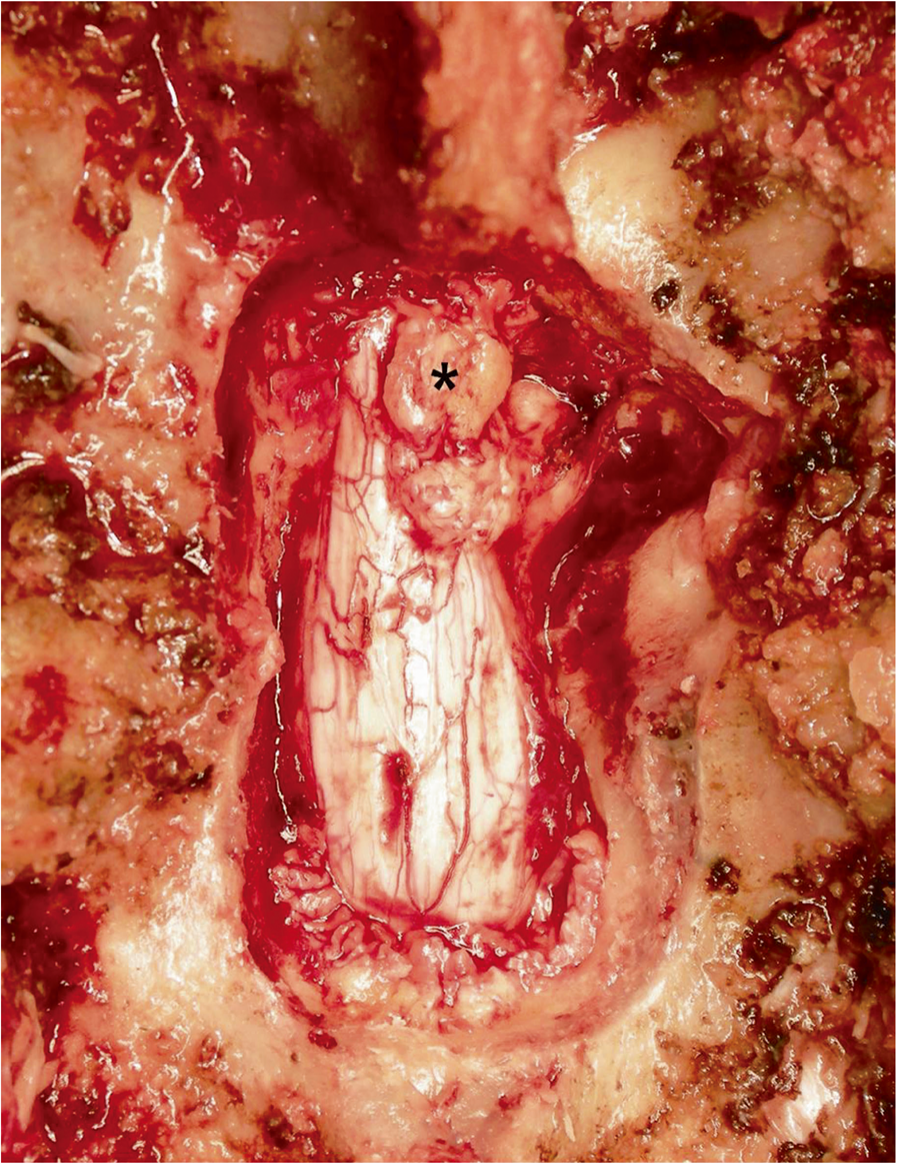
**Intra-operative photograph. **The posteriorly migrated extradural disc fragment after laminectomy is apparent (asterisk).

The patient underwent emergency surgery consisting of laminectomy at T9-T10 with right medial facetectomy, removal of the mass lesion, and posterior instrumented fusion. The mass, which was not adherent to the dural sac, connected with the T9-T10 intervertebral disc space (Figure 2). The intra-operative diagnosis was thus a posteriorly migrated thoracic disc herniation. Histological examination of the surgical specimen revealed a degenerated cartilaginous mass consistent with sequestered intervertebral disc material. 

The patient had an uneventful post-operative course, with clear improvements in lower-extremity strength and urinary continence. He was able to resume walking without a cane by 4 weeks after surgery. At the most recent follow-up, 2.5 years later, he had no residual signs or symptoms.

## Discussion

Thoracic disc herniation is far less common than cervical or lumbar disc herniation. Only 0.15% to 4% of all symptomatic disc herniations occur in the thoracic spine [6]. Posteriorly sequestrated disc herniation is rare but is sometimes reported in the lumbar spine [7]. However, to the best of our knowledge, only five cases of posteriorly migrated thoracic disc herniation have been reported in the English-language literature [1-5]. All reported patients with posteriorly migrated thoracic disc herniation, including our patient, were men with a mean age at onset of 54.7 years (range, 47 to 66 years) [1-5]. Our patient and previously reported patients all showed a disc fragment with iso- to hypointense signals on T1-weighted imaging scans, slight signal hyperintensity on T2-weighted imaging studies, and peripheral contrast enhancement [2,4].

This entity should be considered among the differential diagnoses for an enhancing posterior extradural mass in the thoracic spine [4]. Differential diagnoses of a posterior epidural mass lesion, particularly a rim-enhancing epidural mass, in the thoracic spine include epidural abscess, epidural hematoma, synovial cysts, and extradural neoplasms. Although pre-operative differential diagnosis is important, definitive diagnosis of posterior epidural migration of thoracic disc herniation using MRI alone is unlikely because of the extreme rarity of this pathology.

Clinical information, including sudden onset with normal laboratory findings, may help in reaching a diagnosis. Five of six patients (including ours) with posteriorly migrated thoracic disc herniation presented with sudden onset of severe motor weakness [1-5]. Generally, onset of neurological complications from neoplasm or synovial cysts is gradual, and absence of medical history or abnormal laboratory data is inconsistent with a diagnosis of epidural abscess or hematoma. Spontaneous hematoma may occur without abnormal laboratory data, but early-phase hematoma does not usually demonstrate enhancement [2]. A rim-enhancing lesion associated with hematoma is usually seen in its resolving state [2].

## Conclusions

Herein we report the case of a patient with an extremely rare presentation of posteriorly migrated thoracic disc herniation. The patient experienced sudden onset of incomplete paraplegia, and surgical treatment relieved the symptoms. Pre-operative diagnosis of posterior epidural migration of a herniated thoracic disc based on MRI alone may be difficult, given the rarity of this pathology. However, this entity should be considered among the differential diagnoses for an enhancing posterior thoracic extradural mass.

## Consent

Written informed consent was obtained from the patient for publication of this case report and any accompanying images. A copy of the written consent is available for review by the Editor-in-Chief of this journal.

## Competing interests

The authors declare that they have no competing interests.

## Authors’ contributions

The operation was performed by NM. All authors were involved in the writing of the manuscript and the patient’s clinical care. All authors read and approved the final manuscript.
